# Periodic density functional theory calculations of bulk and the (010) surface of goethite

**DOI:** 10.1186/1467-4866-9-4

**Published:** 2008-05-13

**Authors:** James D Kubicki, Kristian W Paul, Donald L Sparks

**Affiliations:** 1 Dept. of Geosciences and the Earth & Environmental Systems Institute, The Pennsylvania State University, University Park, PA 16802, USA; 2 US Army Research Laboratory, AMSRD-ARL-WM-BD, APG, MD 21005, USA; 3 Department of Plant and Soil Sciences, 152 Townsend Hall, University of Delaware, Newark, DE 19716, USA

## Abstract

**Background:**

Goethite is a common and reactive mineral in the environment. The transport of contaminants and anaerobic respiration of microbes are significantly affected by adsorption and reduction reactions involving goethite. An understanding of the mineral-water interface of goethite is critical for determining the molecular-scale mechanisms of adsorption and reduction reactions. In this study, periodic density functional theory (DFT) calculations were performed on the mineral goethite and its (010) surface, using the Vienna *Ab Initio *Simulation Package (VASP).

**Results:**

Calculations of the bulk mineral structure accurately reproduced the observed crystal structure and vibrational frequencies, suggesting that this computational methodology was suitable for modeling the goethite-water interface. Energy-minimized structures of bare, hydrated (one H_2_O layer) and solvated (three H_2_O layers) (010) surfaces were calculated for 1 × 1 and 3 × 3 unit cell slabs. A good correlation between the calculated and observed vibrational frequencies was found for the 1 × 1 solvated surface. However, differences between the 1 × 1 and 3 × 3 slab calculations indicated that larger models may be necessary to simulate the relaxation of water at the interface. Comparison of two hydrated surfaces with molecularly and dissociatively adsorbed H_2_O showed a significantly lower potential energy for the former.

**Conclusion:**

Surface Fe-O and (Fe)O-H bond lengths are reported that may be useful in surface complexation models (SCM) of the goethite (010) surface. These bond lengths were found to change significantly as a function of solvation (i.e., addition of two extra H_2_O layers above the surface), indicating that this parameter should be carefully considered in future SCM studies of metal oxide-water interfaces.

## Introduction

Goethite (α-FeOOH) is a common and reactive mineral in the environment [1]. α-FeOOH is the most thermodynamically-stable form of the Fe-oxyhydroxides found in soils, groundwater, and acid mine drainage precipitates [2]. α-FeOOH is an excellent adsorbent of contaminants (e.g., arsenate) and nutrients (e.g., phosphate) and is an electron receptor for anaerobic bacterial respiration under anoxic conditions ([3,4] and references therein). Consequently, the surface chemistry of α-FeOOH is important for understanding many environmental processes. One of the most stable and well-studied low-index surfaces of α-FeOOH is the (010) surface (*Pbnm *space group = (100) in the *Pnma *space group; [5,6]). As a result, this study will focus on the (010) α-FeOOH surface.

**Figure 1:**
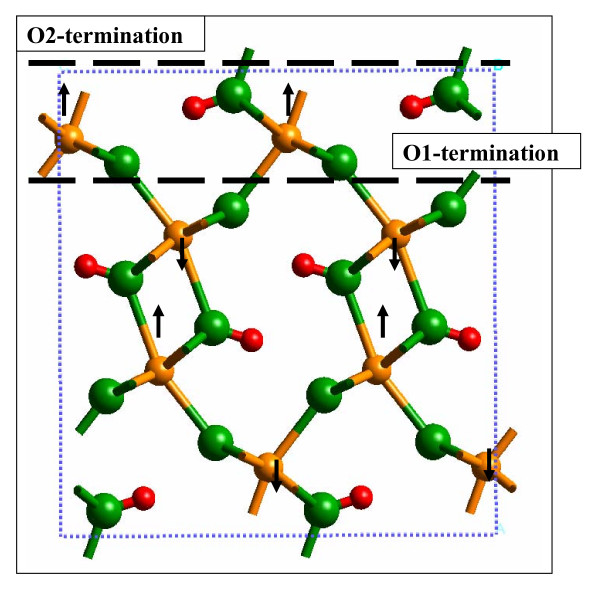
**Possible terminations of the (010) α-FeOOH surface in the *Pbnm *space group (or (100) surface in the *Pnma *space group) are the O1-termination defined between non-protonated O atoms and the O2-termination defined between the protonated O atoms.** Rakovan et al. [5] have determined that the O2-termination is the stable termination, so this was the termination selected for this study.

Several molecular modeling studies have been published related to α-FeOOH and goethite-H_2_O interfaces. For example, Rosso and Rustad [7] published local density approximation (LDA) and generalized gradient approximation (GGA) density functional theory (DFT) calculations on the structures of diaspore, goethite, boehmite, lepidocrocite, akaganeite, guyanaite and grimaldiite that reproduced observed structures of these minerals to within 3%. Rustad et al. [8] used a force field that allowed for the dissociation of H_2_O, in order to perform classical molecular dynamics (MD) simulations of the (110) α-FeOOH-H_2_O interface. Rakovan et al. [5] combined atomic force microscopy, X-ray photoelectron spectroscopy (XPS), low-energy electron diffraction, and periodic molecular orbital calculations, and proposed that the most stable termination of the (010) α-FeOOH surface was the O2-termination. In the O2-termination, the surface is cleaved between protonated O atoms rather than the O1 non-protonated O atoms of the bulk structure (Fig. 1). Shroll and Stratsmaa [9] performed classical MD simulations with an AMBER-type force field to examine goethite-water interactions and found a mineral influence on the water structure out to 5.5 Å. Kerisit et al. [6] employed a polarizable version of the SPC/E water model, along with a α-FeOOH-H_2_O force field [10], to describe the structure of electrolyte solutions near the (010) α-FeOOH surface. Most recently, Aquino et al. [11] performed DFT and Møller-Plesset calculations to investigate the H-bonding of H_2_O to clusters of the (110) α-FeOOH surface.

The calculations reported here are different from these previous studies in that they are periodic DFT energy minimizations of bulk goethite and the (010) α-FeOOH-water interface. The use of quantum chemistry to model the surface distinguishes this work from previous classical simulations. Furthermore, the use of periodic boundary conditions separates these results from those of Aquino et al. [11]. Energy minimizations performed using periodic DFT can be used to verify the accuracy of the computational approach before expending dramatically more computational resources to perform DFT-based MD simulations of the α-FeOOH-water interface.

The purposes of this study were to test the ability of periodic DFT to:

(1) reproduce the structures and vibrational frequencies of bulk α-FeOOH,

(2) predict the surface bond distances for use in surface complexation models, and

(3) investigate the H-bonding of H_2_O at the α-FeOOH-H_2_O interface.

Fe-bearing minerals present a significant challenge for DFT calculations because the electronic ground-state of Fe is typically in a high-spin state. Furthermore, α-FeOOH is anti-ferromagnetic and the spin states of individual Fe atoms within a model must be carefully controlled. Once the DFT methodology, as implemented in VASP, can be shown to reliably reproduce static observables, such as crystal structures and vibrational frequencies, then similar computational methods can be employed to perform quantum MD simulations of reactions at the mineral-H_2_O interface.

## Methods

Bulk α-FeOOH and the (010) surface models were built using the Crystal Builder and Surface Builder modules of Cerius^2 ^4.9 [12], respectively. The α-FeOOH lattice parameters and initial atomic positions were based upon the previously published experimental X-ray diffraction measurements of Szytula et al. [13]. It is imperative to note that the Cerius^2 ^4.9 program uses the *Pnma *space group for α-FeOOH. In the *Pbnm *space group the (010) surface, which is commonly used for α-FeOOH [13], is the (100) surface in the *Pnma *space group (P.J. Heaney, pers. comm.).

The (010) α-FeOOH surface employed in this study was cleaved from the (100) plane of α-FeOOH in the *Pnma *space group with the O2-termination (Fig. 1). This surface structure is consistent with that of Kendall et al. [14]. The 2-D periodic slab generated by this process was stoichiometric, neutral, and symmetric. The bare surface consisted of bridging OH groups (Fe_2_OH or μ-hydroxyl), bridging oxo groups (Fe_3_O or μ_3_-oxo), and 5-fold coordinated Fe atoms. Thus, to hydrate the surface and keep it neutral, H_2_O molecules were coordinated to the surface Fe atoms to fulfill octahedral coordination. In this configuration, the hydrated model was neutral and the presence of Fe-OH_2 _functional groups did not indicate a positively-charged surface of protonated Fe-OH functional groups, as is commonly assumed for metal oxide surfaces. The question of whether or not H_2_O favorably dissociates on this surface was also examined in this study. The O2 atoms are unlikely to accept a second proton (i.e., form an Fe_2_OH_2 _^+ ^site), so if the (Fe)-OH_2 _groups were to donate a proton it would be transferred to the O atoms bonded to three Fe atoms just below the α-FeOOH-water interface (i.e., Fe_3_O + H^+ ^→ Fe_3_OH^+^). The relative potential energies of these two configurations were compared. If the O1-termination were used, then dissociation would be more probable because the 5-coordinate surface Fe atoms could adsorb an H_2_O molecule and this could dissociate to form Fe-OH and protonated O1 surface atoms (i.e., FeOH_2 _+ FeO → 2FeOH). However, since Rakovan et al. [5] determined that this termination was less stable, we did not model it in our study.

All calculations were performed with the Vienna *Ab Initio *Simulation Package (VASP; [15]). The projector-augmented wave (PAW) method [16,17] was used in combination with the Perdew, Burke, and Ernzerhof (PBE) [18] exchange-correlation functional. The O_h and H_h pseudopotentials, as implemented in VASP, were used for the O and H atoms, respectively. The Fe_pv pseudopotential, which includes 14 electrons in the valence shell and treats the 3-p electrons explicitly, was used for the Fe atoms. The plane-wave kinetic energy cutoff was set to 700 eV. (Note: Softer pseudopotentials and a lower energy cutoff were tested and resulted in much poorer reproduction of the observed goethite crystal structure.) Energy differences of 1 × 10^-4 ^eV/atom and energy difference gradients of -0.02 eV/Å were used as convergence criteria. The number of unpaired electrons for the Fe atoms were set to five (i.e., high-spin d^5 ^electronic configuration), with alternating (010) planes in positive and negative spin directions (Fig. 1; [19]). This ensured that the model mimicked the anti-ferromagnetic ground-state of α-FeOOH and that the overall magnetization remained close to zero.

Two bulk α-FeOOH simulation cells were used, namely a 1 × 1 × 1 unit cell model and a 1 × 3 × 2 supercell model. P1 symmetry was applied in both cases. The Monkhorst-Pack [20] scheme was used to generate the k-point sampling grids within the Brillouin zone. For the bulk calculations, a 2 × 6 × 4 grid was used for the 1 × 1 × 1 unit cell model because the unit cell was approximately 9.9 × 3.0 × 4.6 Å. For the 1 × 3 × 2 supercell model, a 1 × 1 × 1 grid (i.e., gamma-point) was used because the simulation cell was nearly cubic with dimensions of 9.9 × 9.0 × 9.2 Å. This combination of direct and reciprocal lattices provided nearly isotropic accuracy with respect to the energy calculations [21,22]. Any difference in the accuracy of the energy between small and large simulation cells is not significant because we only compared calculated structures and not the energies between different models. The "Accurate" precision level (which uses the energy cut-off input as 700 eV and doubles the number of grid points in the fast Fourier transforms used to describe the charge density), as implemented in VASP, was used in all cases. Each k-point grid for sampling the Brillouin zone utilized a first-order Gaussian smearing function [23] of width sigma = 0.1 eV (i.e., ISMEAR = 0 and SIGMA = 0.1 in the VASP input file) in each calculation.

Variable-cell energy minimizations were performed for the calculations of bulk α-FeOOH, but only the atomic positions were allowed to relax for the energy minimizations of the (010) α-FeOOH surfaces. Vibrational frequencies were calculated for the energy-minimized bulk and surface slab models, using a finite difference method (i.e., each atom is displaced individually around its equilibrium position by approximately 0.1 Å) and numerical solution of the Hessian matrix. This solution assumes a harmonic oscillator, so any anharmonicity that is present in the actual vibrational modes will not be accounted for. This is especially important for O-H stretching modes with stronger H-bonds. Energy minimizations and frequency calculations were performed with both the SP-GGA and SP-GGA+U methods in most cases (only the extraordinarily long frequency calculations for the larger models were not repeated with SP-GGA+U). In both cases, the GGA functional corresponded to the SP-PBE exchange-correlation functional. Dudarev's rotationally invariant approach to the SP-GGA+U method was used here [24]. The effective on-site Coulomb and exchange interaction parameters for each Fe atom were set to 4 eV and 1 eV, respectively, as recommended by Rollmann et al. [25]. See Rollmann et al. [25] for details on the SP-GGA+U method applied to α-Fe_2_O_3_.

Periodic slabs with surface areas of 13.86 Å^2 ^(1 × 1) and 124.76 Å^2 ^(3 × 3) were used for the surface model calculations to test the effect of increasing the surface area on the predicted structures. Slab thickness was 8.88 Å in both cases. Vacuum gaps with z-dimension of 10 Å were used to separate the slabs. Solvated models were built by adding one H_2_O molecule per 5-coordinate surface Fe atom with a bond distance of approximately 2.1 Å (2 H_2_O molecules in the 1 × 1 and 18 H_2_O molecules in the 3 × 3 models). The H_2_O molecules were then relaxed using the COMPASS force field [26] while all of the goethite slab atoms were fixed. For the hydrated models, these were filled with H_2_O molecules to approximate a density of 1 g/cm^3 ^as closely as possible. This gave a total of 8 and 54 H_2_O molecules in the 1 × 1 and 3 × 3 models, respectively. This represents three layers of hydration (L1 – directly bound to the surface Fe atoms, L2 – H-bonding to the L1 layer, and L3 – H-bonding to the L2 layer; see [27] for experimental verification of these layers on TiO_2 _and SnO_2 _surfaces). The H_2_O molecules were initially positioned to optimize their H-bonding with the surface. An orientation that allowed the H_2_O to act as an H-bond donor to the O2 surface atoms and H-bond acceptor from the surface H_2_O molecules was selected. This initial configuration has not been verified experimentally and will influence the final results of the energy minimizations. Molecular dynamics simulations should be performed to test the reliability of this initial configuration. However, we justify this current decision based on the acidity of the surface groups present. The H^+ ^ions in the Fe-OH_2 _surface groups should be more acidic than the H^+ ^ions on the O2 surface groups [28]; hence the Fe-OH_2 _sites should be better H-bond donors and the O2 atoms better H-bond acceptors ([29] and references therein).

## Results

### Bulk structure and vibrational frequencies

**Figure 2:**
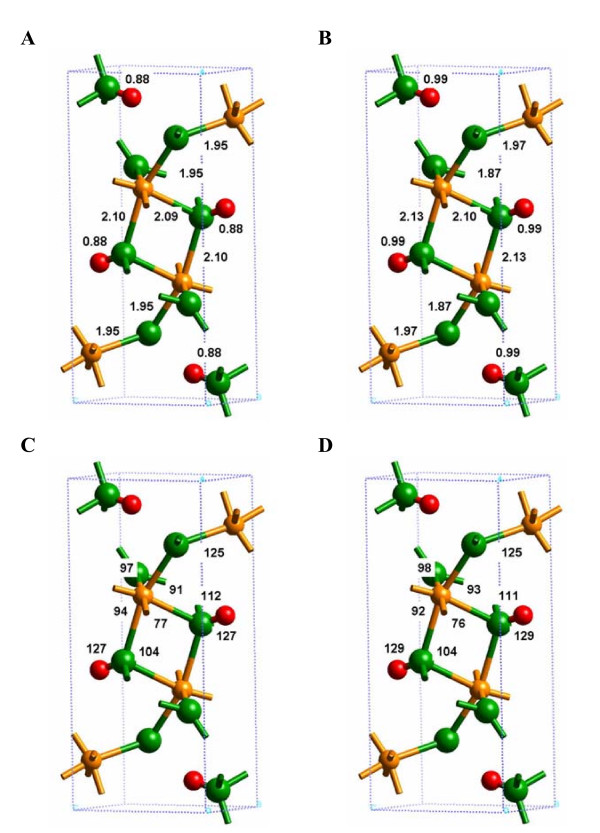
**Comparison of the bulk Fe-O(H), (Fe)O-H and H---O distances from observation (A) and the VASP calculations (B), using hard pseudopotentials.** Note that tests performed using soft pseudopotentials for O and H resulted in much poorer agreement with experiment. Observed (C) and calculated (D) angles.

**Figure 3:**
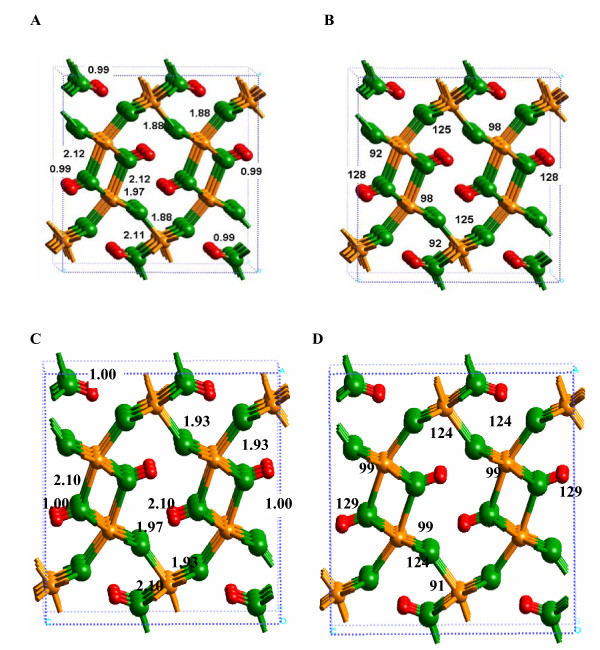
**1 × 3 × 2 supercell calculation of the bulk α-FeOOH structural distances (A) and angles (B) with SP-GGA and (C,D) SP-GGA+U.** Relatively small changes in the interatomic distances and angles were predicted with the correction for self-interaction of Fe d-orbitals.

The first test of the computational methodology was to compare the observed and calculated crystal structures. The results in Table 1 show that both the 1 × 1 × 1 unit cell (Additional file 1) and the 1 × 3 × 2 supercell (Additional file 2) models reproduced the observed lattice parameters and fractional coordinates, within a small percentage error. The current results decrease the already small discrepancy between the calculated values of Rosso and Rustad [7] and the observations of Szytula et al. [13]. For example, the a, b and c lattice parameters in [7] are 9.80, 3.00 and 4.49 Å compared to the 9.95, 3.00 and 4.60 Å computed in this study. Hence, the maximum error is decreased from 3% to 0.5% via use of the pseudopotentials employed in this study. Compared to the unit cell, the supercell methodology provided a more stringent test for predicting the observed crystal structure because there was more flexibility for the ions to relax from their initial positions. Figures 2 and 3 graphically illustrate the comparison between the observed and calculated crystal structures. The agreement was good and did not significantly depend on whether the 1 × 1 × 1 unit cell or the 1 × 3 × 2 supercell was used for the DFT calculation. Differences were on the order of a few percent in each case. The SP-GGA+U method (Additional file 3) gave the same lattice parameters as calculations without the +U correction although the fractional coordinates were slightly different between the two methods (Table 1). These results suggest that the structures predicted with the methodology described above are accurate and independent of the size of the model system for the bulk α-FeOOH mineral.

**Table 1:** Comparison of observed [44] and calculated lattice parameters and atomic positions for bulk α-FeOOH.

	**Experimental**	**Calc1**	**Calc2**	**Calc3**
a	9.95	9.94	9.95	9.95
b	3.01	2.99	3.00	3.00
c	4.62	4.60	4.60	4.60
α = β = γ	90	90	90	90
O1	.05, .75, .20	.05, .75, .20	.05, .75, .20	.06, .75, .20
O2	.20, .75, .71	.20, .75, .71	.20, .75, .70	.20, .75, .68
O3	.30, .25, .22	.30, .25, .21	.30, .25, .20	.30, .25, .19
O4	.45, .25, .70	.45, .25, .70	.45, .25, .70	.44, .25, .70
O5	.55, .75, .30	.55, .75, .30	.55, .75, .30	.56, .75, .30
O6	.70, .75, .78	.70, .75, .79	.70, .75, .80	.70, .75, .82
O7	.80, .25, .28	.80, .25, .29	.80, .25, .30	.80, .25, .32
O8	.95, .25, .80	.95, .25, .80	.95, .25, .80	.94, .25, .80
Fe1	.15, .25, .95	.15, .25, .96	.15, .25, .96	.15, .25, .94
Fe2	.35, .75, .45	.35, .75, .46	.35, .75, .46	.35, .75, .44
Fe3	.65, .25, .55	.65, .25, .54	.65, .25, .54	.65, .25, .56
Fe4	.85, .75, .05	.85, .75, .04	.85, .75, .05	.85, .75, .06
H1	.10, .75, .40	.08, .75, .41	.08, .75, .40	.09, .75, .40
H2	.40, .25, .90	.42, .25, .91	.42, .25, .90	.41, .25, .90
H3	.60, .75, .10	.58, .75, .09	.58, .75, .09	.59, .75, .10
H4	.90, .25, .60	.92, .25, .59	.92, .25, .59	.91, .25, .60

Calc1 = 1 × 1 × 1 unit cell; Calc2 = 1 × 3 × 2 supercell; Calc3 = 1 × 3 × 2 supercell with SP-GGA+U correction.

One significant discrepancy between the observed and calculated structural parameters was the (Fe)O-H bond length. Experimentally, the (Fe)O-H bond length is 0.88 Å, whereas the DFT-calculated (Fe)O-H bond length was approximately 0.99 Å. Given that the IR frequencies and intensities of Fe-OH vibrational modes strongly depend upon O-H bond lengths [30], a difference of 0.1 Å is expected to be significant. Other calculations, such as molecular orbital theory calculations, also predict O-H bond lengths in the range of 0.96 to 1.00 Å in minerals and Fe-hydroxide molecular clusters [31-34]. The vibrational frequencies predicted by molecular clusters are generally in good agreement with observation for a range of compounds and materials [35]. Furthermore, X-ray diffraction methods are insensitive to the positions of H atoms in crystalline materials and thus their atomic positions must be inferred. Consequently, it is possible that the calculated (Fe)O-H bond length is more accurate than the experimentally estimated (Fe)O-H bond length.

The reproduction of crystal structures can test the ability of a particular method to determine a system's minimum energy position on its potential energy surface. Vibrational frequencies probe the second derivatives of the potential energy surface (i.e., the Hessian matrix) around this minimum and are related to the force constants of bonds. Atomic vibrations can also be used to calculate entropies and other thermodynamic properties of crystals [36]. Consequently, vibrational frequencies are useful observables to calculate and serve to further validate a computational methodology.

Table 2 lists the observed and calculated vibrational frequencies for α-FeOOH. In general, there is good correspondence between the calculated and observed vibrational frequencies, especially considering that the DFT-calculated frequencies are determined numerically within the harmonic oscillator approximation. Use of the SP-GGA+U method decreases the calculated O-H stretching frequencies by approximately 200 cm^-1 ^compared to the SP-GGA method and brings the calculated values closer to observed frequencies (Table 2). Whether or not this apparent agreement is the result of improved H-bond modeling is not clear because we do not know the percent anharmonicity in these O-H vibrations. However, if we consider the difference between calculated and observed values to be solely due to anharmonicity, the SP-GGA results predict an anharmonicity of approximately 8% whereas the SP-GGA+U method predicts approximately 3% anharmonicity. The latter value is more consistent with previous DFT results comparing observed and predicted vibrational frequency anharmonicities [37], so we believe the SP-GGA+U method is more accurate.

**Table 2:** Comparison of observed (Expt 1 = [45]; Expt 2 = [38]) and calculated IR frequencies for bulk α-FeOOH.

**Mode**	**Calc^a^**	**Calc^b,c^**	**Calc^d^**	**Expt 1**	**Expt 2**
**cm^-1^**	**(cm^-1^)**	**(cm^-1^)**	**(cm^-1^)**	**(cm^-1^)**	**(cm^-1^)**

O-H	3416	3400	3227	3140	3200
O-H	3411	3391	3217		
O-H	3404	3385	3213		
O-H	3396	3373	3206		
δ(OH)	954	974	1011		1000
δ(OH)	930	966	1007		
δ(OH)	901	958	958		
δ(OH)	896	911	956		
γ(OH)	839	857	934	893	887
γ(OH)	810	848	896		
γ(OH)	806	830	892		
γ(OH)	797	819	888	793	
Fe-O_s_	599	576	640	620	610
Fe-O_s_	547	567	623		
Fe-O_s_	525	558	540		
Fe-O_s_	482	547	538		
δ(Fe-O-Fe)	467	477	526	495	
δ(Fe-O-Fe)	424	461	481		
δ(Fe-O-Fe)	417	453	459		
δ(Fe-O-Fe)	395	441	442		
Fe-O_as_	391	426	410	405	
Fe-O_as_	376	413	405		
Fe-O_as_	371	397	401		
Fe-O_as_	357	413	399		
O lattice	348	379	396		
O lattice	345	372	395		
O lattice	343	365	380		
O lattice	321	361	375		
Fe-O-Fe	309	354	360		
Fe-O-Fe	293	338	334		
Fe-O-Fe	284	328	307		
Fe-O-Fe	268	314	301		
Fe-O-Fe	258	309	293		
Fe-O-Fe	254	301	288		
Fe-O-Fe	242	287	270		
Fe-O-Fe	228	274	266		
Fe---Fe	223	262	255		
Fe---Fe	211	246	241		
Fe---Fe	179	243	237		
Fe---Fe	174	240	206		
FeO_6 _rock	143	230	185		
FeO_6_	108	210	163		
FeO_6_	78	177	132		
FeO_6_	69	164	117		
Tunnel Distortion	24	143	---		

a = 1 × 1 × 1 unit cell; b = 1 × 3 × 2 supercell; c = average of 6 frequencies; d = 1 × 1 × 1 unit cell with SP-GGA+U.

VASP 4.5 does not calculate the IR intensities of the vibrational modes. Thus, it is difficult to assign calculated frequencies unequivocally to observed vibrational modes. Because there are a large number of calculated vibrational frequencies, correlations between model and observed frequencies may be fortuitously accurate. However, given these caveats, the DFT-calculated displacements in Table 2 reasonably correspond to experimentally-measured vibrational modes both from IR spectroscopy and inelastic incoherent neutron scattering [38]. Therefore, we can be confident that the calculations are reasonably modeling the potential energy surface around the minimum energy structure.

### Surface structure, H-bonding and vibrational frequencies

#### Hydrated surface

**Figure 4:**
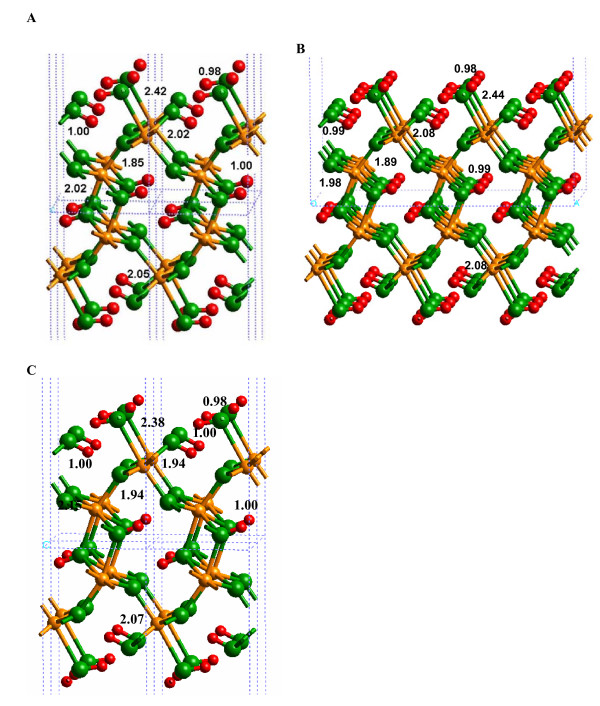
**Calculated structures of the hydrated (010) α-FeOOH surface, using (A) 1 × 1 and (B) 3 × 3 slabs, which showed that significant relaxation of the Fe-O bonds occurred at the surface compared to the bulk (Fig. 3a).** System size effects were minimal for this model system. (C) Shows the 1 × 1 slab structure with SP-GGA+U included, and the bond lengths change by a few hundredths of an Angstrom compared to Fig. 4a.

After cleaving the (010) α-FeOOH surface, H_2_O molecules were added to each 5-fold coordinated Fe atom (Fig. 4). Unless metal oxides are cleaved under ultra-high vacuum, gaseous H_2_O will adsorb at the surface to satisfy under-coordinated metal atoms. The layer of coordinated H_2_O molecules in this study was analogous to the L1 layer observed by Mamontov et al. [27] for TiO_2 _and SnO_2_. We term this state a "hydrated" surface to distinguish it from a bare surface without adsorbed H_2_O and the "solvated" surface with at least two H_2_O layers H-bonded to the hydrated surface. The structures of the hydrated surface models are displayed in Figure 4 (Additional files 4–6). Regardless of whether the 1 × 1 or 3 × 3 slabs were used in the DFT calculations, the DFT-calculated bond lengths were similar to within a few hundredths of an Angstrom (Fig. 4a versus Fig. 4b). However, changes in the interatomic distances can be on the order of 0.1 Å when this surface was modeled with the SP-GGA+U method compared to SP-GGA (e.g., compare Fig. 4a and 4c). The SP-GGA+U method appears to predict O-H and H-bonding more realistically based on the bulk goethite results, so values in Fig. 4c are likely to be more accurate. Because it was impractical to perform a frequency calculation for the 3 × 3 slab using our currently available computational resources, we focus on the 1 × 1 slab model (Fig. 4a).

The calculated Fe-O(H_2_) bond length was significantly longer (Fig. 4) than the Fe-O bond it replaced in the bulk structure (2.42 versus 1.87 Å; Fig. 2b, Fig. 3a or 2.38 versus 1.93 for the SP-GGA+U results, Fig. 3c versus 4c). Furthermore, the surface Fe-O bonds shortened from approximately 2.13 to 2.02 Å to compensate for the added H_2_O molecules, as would be predicted by Pauling's rules. A small difference was predicted between the O-H bond lengths of the adsorbed H_2_O molecules and the OH functional groups that occupy the (010) α-FeOOH surface. The former were 0.98 Å and the latter were 1.00 Å due to differences in H-bonding. For example, the adsorbed H_2_O was only weakly H-bonded to the O atom of the Fe-(OH)-Fe linkage (H---O = 2.25 Å and O-H---O = 140°), while the α-FeOOH OH functional groups formed moderately strong H-bonds (H---O = 1.82 Å and O-H---O = 175°). It should be noted that a shorter H---O bond distance and more linear O-H---O angle both correspond to a stronger H-bond [30]. Although this difference may seem insignificant, it has an influence on how solvating H_2_O molecules interacted with the surface, as discussed below.

The DFT-calculated IR vibrational frequencies for the hydrated (010) α-FeOOH surface generally agreed well with observation (Table 3). The agreement is noteworthy because significant error is expected due to the fact that harmonic frequencies are calculated whereas anharmonic frequencies are observed. One exception was the observed vibrational mode near 800 cm^-1^, which had its closest corresponding DFT-calculated frequency at 727 or 758 cm^-1 ^for the SP-GGA and SP-GGA+U results, respectively (Table 3). In addition, the O-H stretching frequencies of the model were predicted, in general, to have higher energies than the observed vibrational mode at 3160 cm^-1 ^(although this error is less in the SP-GGA+U calculation, Table 3). Because the frequencies of the O-H vibrational modes are highly dependent upon the H-bonds they form, this difference was probably due to the presence of more than one layer of adsorbed H_2_O on the (010) α-FeOOH surface. The model O-H functional group with the strongest H-bond, for example, had a corresponding DFT-calculated vibrational frequency of 3210 cm^-1 ^– 50 cm^-1 ^or less than 2% deviation from experiment. This suggestion is tested below in discussing the solvated α-FeOOH surface.

**Table 3:** Comparison of observed and calculated IR frequencies for the hydrated α-FeOOH surface.

**Mode**	**Calc^a^**	**Calc^b^**	**Experimental**
	**(cm^-1^)**	**(cm^-1^)**	**(cm^-1^)**

Surface O-H	3673^c^	3686^c^	3660^d^
Bulk+Surface O-H	3596	3614^c^	3500^d^
Bulk+Surface O-H	3672		
Bulk+Surface O-H	3276^c^	3239^c^	3200^d^
Bulk+Surface O-H	3227	3225^c^	3160^d,e,f^
Bulk+Surface O-H	3057		
δ(HOH)	1578^c^	1574^c^	
δ(OH)	1044	1023	1120^g^
δ(OH)	1003	1019	
δ(OH)	990	995	
δ(OH)	932	955	
δ(OH)	920	944	
δ(OH)	907	939	900^h^
δ(OH)	880	916	885^i^
δ(OH)	761	892	880^h^
δ(OH)	710	758	790^g^, 797^h^, 805^h^
δ(OH)	669	719	
δ(OH)	660	652	
Fe-O+ δ(OH)	654	646	620 (sh)^g^
Fe-O+ δ(OH)	576	622	
Fe-O+ δ(OH)	564	616	

Frequencies less than 600 cm^-1 ^have not been included, but the output file is available as supplemental information.

a = 1 × 1 unit cell

b = 1 × 1 with SP-GGA+U

c = average of two closely spaced frequencies

d. [46]

e. [47]

f. [48]

g. [49]

h. [50]

i. [51]

#### Solvated surface

In this section, we first compare the solvated (010) α-FeOOH surface to the hydrated (010) α-FeOOH surface for the 1 × 1 slab discussed above. Second, a comparison of the solvated (010) α-FeOOH surfaces generated via energy minimization of the 1 × 1 and 3 × 3 slabs are compared. Third, the vibrational frequencies of the 1 × 1 solvated (010) α-FeOOH slab are examined in comparison to experimental vibrational spectra. Last, a comparison of the potential energies of the solvated surfaces with associated and dissociated H_2_O is made.

**Figure 5:**
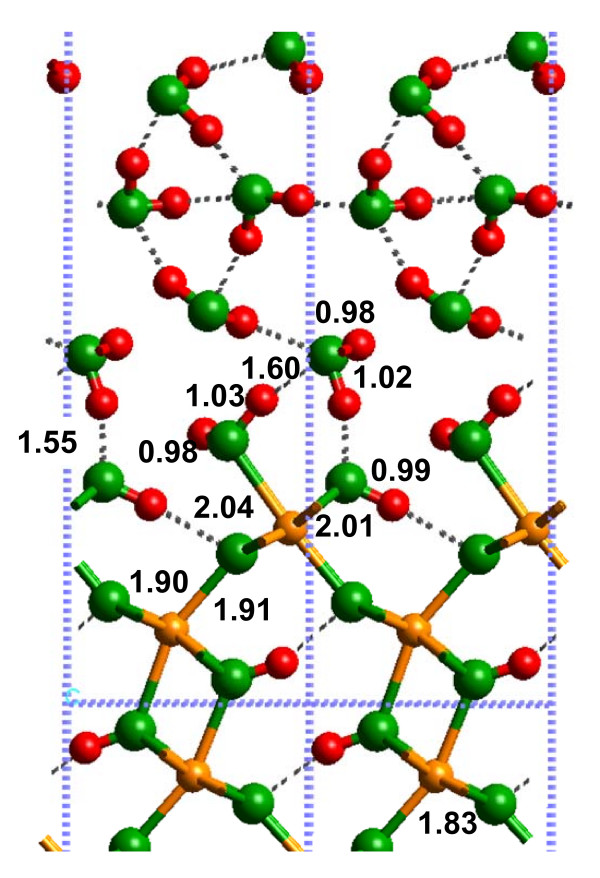
Solvated (010) α-FeOOH surface, using the 1 × 1 surface cell.

The most important difference between the hydrated and solvated (010) α-FeOOH surfaces was the Fe-O(H_2_) bond distance (Fig. 5; Additional file 7). The DFT-calculated Fe-O(H_2_) bond distance changed from 2.42 to 2.04 Å with the addition of two layers of H_2_O molecules H-bonded to the L1 layer (2.38 to 2.02 Å for SP-GGA+U; see Additional file 8). This result indicates that if bond distances predicted via quantum chemical calculations are used to model the mineral-water interface (e.g. [39]), solvation beyond the L1 layer should be considered. For example, metal-oxygen bond distances used in surface complexation models such as the MUSIC model [40] (see Discussion below) will be more accurate if solvent H_2_O molecules are included beyond the L1 layer. Shortening of the Fe-O(H_2_) bond also resulted in lengthening of the remaining Fe-O bonds around the surface Fe atoms, in closer agreement with bulk values (e.g. the 1.85 Å bond in Fig. 4a increased to 1.91 Å in Fig. 5).

H-bonding between the L1 and L2 layers significantly affected the Fe-O bond lengths in the models used in this study. One H_2_O molecule acts as a proton donor and acceptor with the surface forming a 1.60 Å H-bond between (Fe)-OH_2 _functional groups and a 1.55 Å H-bond to the O atom of the Fe-(OH)-Fe surface linkage (Fig. 5). H-bonds can transfer electron density from the O-H bond which allows the O atom in the adsorbed H_2_O to form stronger bonds with Fe(III) on the surface.

**Figure 6:**
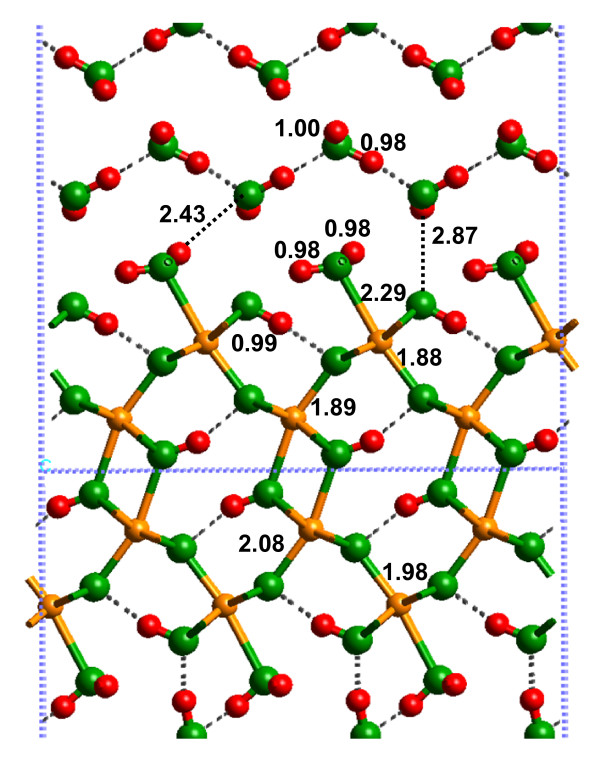
Solvated (010) α-FeOOH surface, using the 3 × 3 slab.

The 1 × 1 slab was highly constrained by symmetry, as evidenced by the changes observed for the energy-minimized 3 × 3 calculation (Fig. 6; Additional file 9). The Fe-O(H_2_) bonds were longer (2.29 Å) for the 3 × 3 slab model, in comparison to the 1 × 1 slab model. However, this bond distance was still not as long as that predicted for the hydrated surface calculations (2.44 Å; Fig. 4b). In the 3 × 3 slab calculation, the L2 layer preferred to create a strong H-bonding network within the L2 layer, rather than to the (010) α-FeOOH surface. H-bonds between the L2 layer and the surface did exist, but these were weak with H---O bond distances of 2.43 Å for (Fe)OH_2_---OH_2 _and 2.87 Å for OH_2_---(OH)Fe_2 _(Fig. 4b). Consequently, a H-bond network between (Fe)-OH_2 _functional groups and Fe-(OH)-Fe linkages was generated, but steric constraints kept these H-bonds fairly weak with H---O distances of 2.3 to 2.5 Å and O-H---O angles of approximately 110°. The only relatively strong H-bonds on the surface were between the Fe-(OH)-Fe hydroxyl groups and the underlying O atoms as discussed for the hydrated surface above.

As mentioned above in the *Hydrated surface *section, the OH functional groups of α-FeOOH were H-bonded to the surface, preventing them from behaving as proton donors to solvent H_2_O molecules. Because the Fe-OH_2 _functional groups only acted as proton donors in H-bonds to solvent H_2_O molecules, a pattern of H_2_O molecules formed in the L2 layer whereby each H_2_O molecule formed a donating H-bond to the O of the Fe-(OH)-Fe group and an accepting H-bond to the Fe-OH_2 _functional group (Fig. 5). The DFT energy minimizations exaggerated this pattern, so MD simulations should be performed to test how stable this structure is at finite temperatures. However, the underlying structure of the mineral should influence the H-bonding pattern of the L2 structure.

As discussed above for the hydrated (010) α-FeOOH surface, the DFT-calculated frequencies in the O-H stretching region had significantly higher energies than the observed vibrational mode near 3200 cm^-1 ^for α-FeOOH (Table 4; [41]). However, this vibrational mode is very broad and the frequencies are strongly affected by H-bonding. With the addition of H_2_O to form the solvated (010) α-FeOOH surface model, the calculated O-H stretching frequencies decreased significantly and ranged from 2370 to 3680 cm^-1 ^– a similar range to the observed IR vibrational mode (Table 4). Deconvoluting this broad vibrational mode and relating frequencies to individual hydroxyl groups was not practical. However, the agreement between observation and model suggests that the calculations can reasonably predict H-bonding at the surface. Furthermore, the DFT results are consistent with the idea that oxides typically adsorb at least two to three layers of H_2_O from the atmosphere that affect the structure and vibrational spectra of metal-oxides.

**Table 4:** Comparison of observed and calculated IR frequencies for the solvated α-FeOOH surface (1 × 1 surface unit cell).

**Mode**	**Calc1**	**Calc2**	**Experimental**
	**(cm^-1^)**	**(cm^-1^)**	**(cm^-1^)**

L2 O-H	3668	3682	3660^a^
δO2-H	3667	3669	
L1 O-H	3663	3664	3590^a ^3160^b,c^
L2 O-H	3647	3641	
L2 O-H	3522	3516	
L2 O-H	3497	3495	3515^a^
L2 O-H	3423	3434	
L1+L2 O-H	3377	3379	3380^a^
L2 O-H	3362	3282	
L1 O-H	3337	3218	
L2 O-H	3319	3185	
δO2-H+L1 O-H	3259	3179	3200^a^
L3O-H	3184	3161	
δO2-H	3127	3061	3160^a^
L1+L2 O-H	3079	2994	3130^a^
L3 O-H	2977	2968	
δO2-H+L3 O-H	2870	2940	
L1 O-H	2779	2928	
L1 O-H	2675	2725	
δO2-H+L3 O-H	2369	2668	
L1 H-O-H	1761	1739	
δO2-H+L3 H-O-H	1660	1655	
δO2-H+L2 H-O-H	1628	1633	
δO2-H+L1 H-O-H	1623	1609	
δO2-H+L3 H-O-H	1610	1606	
δO2-H+L2 H-O-H	1583	1563	
δO2-H+L1 H-O-H	1572	1554	
δO2-H+L3 H-O-H	1567	1546	
δ(OH) L3	1277	1223	
δ(OH) L1	1190	1160	1120^d^
δ(OH) L1	1108	1153	
δ(OH) O2+L1+L2+L3	1051	1546	
δ(OH) O2+L2+L3	1035	1223	
δ(OH) O2+L2	1019	1160	
δ(OH) L2+L3	1000	1152	
δ(OH) L1+L2	991	1143	
δ(OH) O2+L2	958	1087	
δ(OH) L3	949	1083	
δ(OH) L1+L2	941	1071	900^e^
δ(OH) L1+L2	811	1038	880^f^, 885^e^
δ(OH) L1+L2	886	1035	
δ(OH) L2+L3	873	1031	
δ(OH) L2+L3	868	1024	
δ(OH) L2+L3	855	987	
δ(OH) L2+L3	834	967	
δ(OH) L2+L3	821	917	
δ(OH) L1+L2	811	850	805^e^
δ(OH) L1+L2	781	830	790^e^, 797^e^,
δ(OH) L1+L2	726	814	620 (sh)^f^
	688	803	
	665	762	
	640	719	
	629	701	
	613	689	
	603	673	
		635	
		629	
		624	
		621	

Values denoted with an * are the average of two closely-spaced frequencies. Calc1 from the 1 × 1 surface cell SP-GGA and Calc2 from SP-GGA+U results, respectively.

a. [46]

b. [47]

c. [48]

d. [49]

e. [50]

f. [51]

The (010) α-FeOOH surface can form different configurations with respect to H^+ ^positions. The physisorbed H_2_O surface has been discussed above with an Fe_2_OH site from the original bulk structure, an FeOH_2 _terminal group (molecular adsorbed H_2_O) and no H^+ ^on the Fe_3_O sites. Dissociated H_2_O configurations are also possible with H_2_O adding a H^+ ^to the Fe_3_O site to form an Fe_3_OH + Fe_2_OH + FeOH surface (Fig. 5). Theoretically, a surface comprised of Fe_3_OH + Fe_2_O + FeOH_2 _is another combination. The relative energies of these configurations were investigated using the SP-GGA+U method for the solvated surface because this method is thought to result in more accurate energies [25]. (Note: this was not done for the hydrated surface because H-bonding to the L2 likely plays a significant role in stabilizing the protonation state of the surface.) It is also worth noting here that the interatomic distances predicted using the SP-GGA+U method are similar to those obtained for the SP-GGA method.

**Figure 7:**
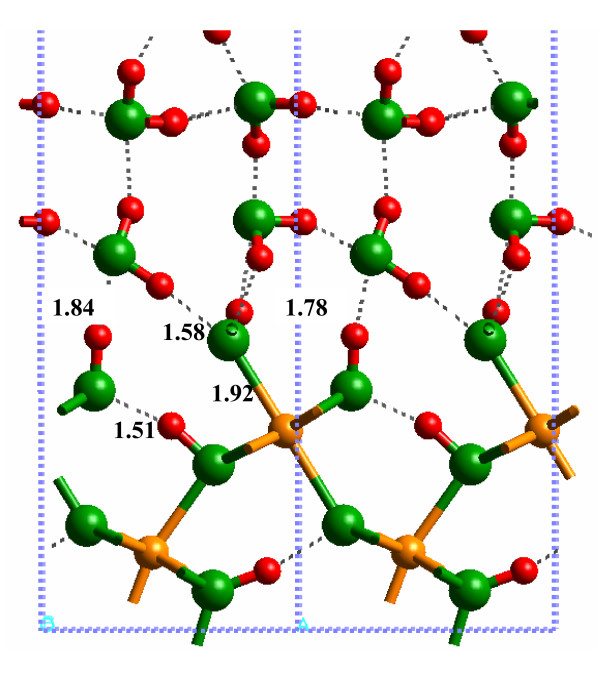
Solvated (010) α-FeOOH surface dissociated (010) with SP-GGA+U.

**Figure 8:**
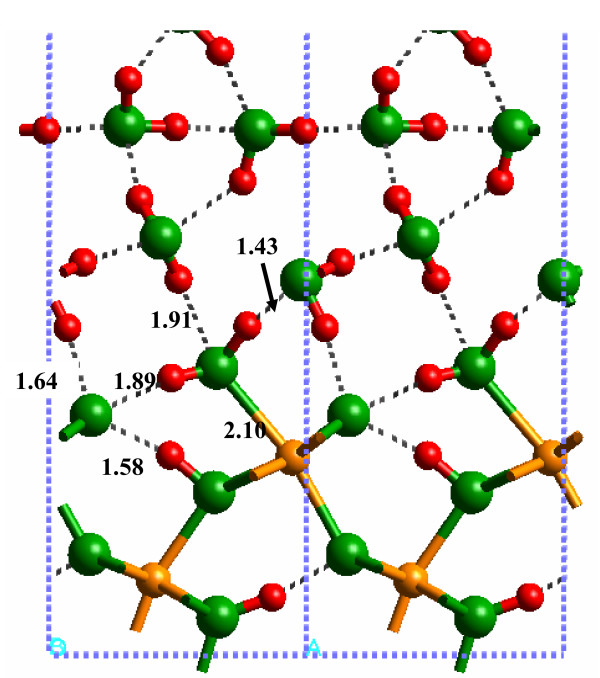
Solvated (010) α-FeOOH surface initial configuration of Fe_3_OH + Fe_2_O + FeOH_2 _with SP-GGA+U.

**Figure 9:**
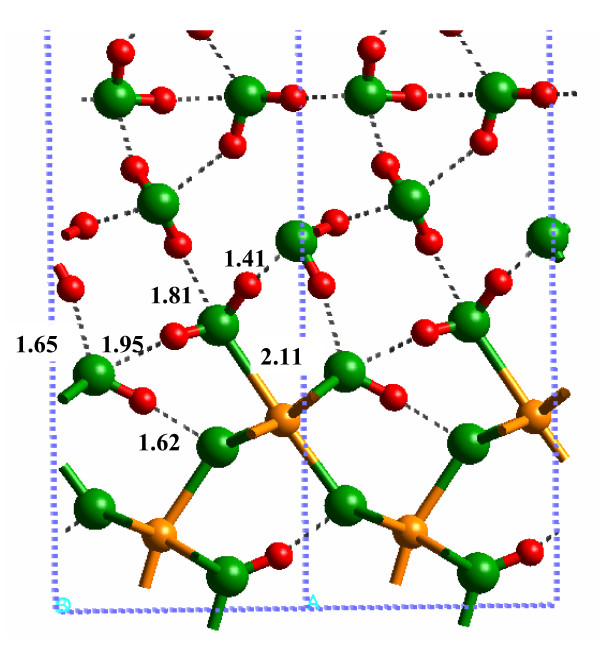
Solvated (010) α-FeOOH surface associated with SP-GGA+U.

A minimum potential energy configuration was found for the dissociated surface with Fe_3_OH + Fe_2_OH + FeOH, as suggested by Rakovan et al. [5] (Fig. 7; Additional file 10). The H-bonding network between the (010) α-FeOOH surface and water, as well as at the surface, shows that all atoms have formed H-bonds. Many of these H-bonds were fairly strong with H---O distances of 1.51 Å between the Fe_3_OH---HOFe_2 _and 1.58 Å between the O atom of the FeOH group and the closest H_2_O molecule. However, the calculated potential energy of this configuration was -213.2039 eV. The Fe_3_OH + Fe_2_O + FeOH_2 _surface (Fig. 8) did not result in a stable potential energy minimum because the Fe_3_OH proton was transferred to form an Fe_2_OH group, resulting in a configuration similar to the original associated surface (i.e., Fe_3_O + Fe_2_OH + FeOH_2 _– Fig. 9; Additional file 11). The calculated potential energy of this configuration was -214.3466 eV or approximately 75 kJ/mol (where the "mol" indicates a mole of H^+ ^on the α-FeOOH (010) surface) lower in energy than the dissociated surface in Fig. 7. Consequently, the current results suggest that the associated surface is likely to be more stable at 25°C unless the entropy of the dissociated surface is greater than 250 J mol^-1 ^K^-1 ^(75 kJ mol^-1^/298K) which is large for a deprotonation reaction [42].

## Discussion

Although the above results represented energy-minimized structures on a neutral surface, and further testing must be performed under various surface charge states with MD simulations, it is instructive to compare the results to previous studies of the goethite-water interface. In our model, we began and ended with a (010) α-FeOOH surface terminated by Fe-OH_2 _functional groups. This is similar to the result of Rustad et al. [8] where Fe-OH_2 _functional groups were generated from their classical MD simulations. However, the initial structure in [8] was a Fe-OH-terminated surface and the Fe-OH_2 _functional groups were created as solvent H_2_O molecules transferred protons to the surface and OH^- ^ions H-bonded to the surface.

A similar discrepancy exists between the current study and the (010) α-FeOOH surface model of Rakovan et al. [5]. In [5], a Fe-OH-terminated surface was the final configuration. Rakovan et al. [5] did not use a solvation layer above their (010) α-FeOOH surface and interpreted their XPS data to conclude that 5-fold coordinated surface Fe atoms should be coordinated with OH functional groups. In our model, such a termination was metastable, but the associated H_2_O surface was lower in potential energy. This is based on a neutral surface that should represent α-FeOOH (010) at the point of zero-charge (PZC). However, the water layers in this model do not contain H^+ ^or OH^-^. Consequently, DFT-based MD simulations would be useful to test whether H^+ ^transfers occur from the surface to solvent H_2_O molecules, resulting in a Fe-OH-terminated surface charge-balanced by H_3_O^+ ^ions or whether a protonated surface and aqueous OH^- ^forms as should occur for this surface, which is below its PZC at pH 7. Simply moving H^+ ^in these energy minimizations could not test this because the H_2_O layers would need to re-arrange significantly to adjust to this H^+^-transfer.

In agreement with a study performed by Kerisit et al. [6], who found ordering of 4 to 5 H_2_O layers, the H_2_O molecules in our solvated model were ordered to at least 3 layers of H_2_O molecules. However, the large degree of ordering in our model structures is partly an artifact of the highly ordered initial state and the fact that energy minimizations at 0 K were performed, not MD simulations at finite temperature. Again, DFT-based MD simulations are necessary to examine the configuration space of the α-FeOOH-H_2_O interface.

The studies discussed above did not report Fe-O(H_2_) bond distances, nor H-bond distances between solvent H_2_O molecules and the α-FeOOH surface, so we cannot compare our DFT-calculated (010) α-FeOOH-H_2_O interface structures to these studies quantitatively. However, Aquino et al. [11] did report O-H bond distances and H-bond distances from their DFT calculations, using cluster models of the (110) α-FeOOH surface. The (110) surface in the study of Aquino et al. [11], however, was terminated by OH functional groups, which complicates comparison. Their O-H bond distances ranged from 0.97 to 1.00 with no systematic variation among the terminal Fe-OH (hydroxo), bridging Fe_2_OH groups (μ-hydroxo), and Fe_3_OH (μ_3_-hydroxo). These values were similar to those predicted in our periodic DFT calculations, but H-bonding played a role in lengthening the Fe_2_OH hydroxyl OH bonds of the hydrated surface compared to terminal Fe-OH_2 _groups in the current work. This effect was not observed for the solvated models.

The range of H-bond distances, 1.6 to 2.3 Å, calculated by Aquino et al. [11] overlaps the range predicted for the solvated 1 × 1 slab of this study. However, as noted above, the H-bond distances were significantly lengthened when the model was expanded to a 3 × 3 surface slab. Consequently, strong DFT-calculated H-bonds were probably an artifact of the system size and the neglect of H-bonding to H_2_O layers above the L2 layer. Qualitatively, this was confirmed by the observation that up to three H-bonds were formed between the α-FeOOH surface and one H_2_O molecule in the Aquino et al. [11] study. On the other hand, such extensive H-bonding was not observed in this study. The closest result obtained in this work to the H-bonding arrangement in [11] is in Fig. 9 where the FeOH_2 _forms both donor and acceptor H-bonds to an H_2_O molecule. The third H-bond is to the O atom of the adjacent Fe_2_OH group, however. Recent work by Nangia et al. [43] has demonstrated the importance of surface H-bonding networks on influencing the structure of the mineral-water interface, so this factor should be considered in models of mineral surfaces.

The MUlti-SIte Complexation (MUSIC) model developed by Hiemstra et al. [39] is useful for predicting the PZC, as well as macroscopic adsorption behavior of mineral surfaces. Consequently, this type of thermodynamic model has great value in geochemistry and environmental chemistry. MUSIC requires charges, metal-oxygen bond lengths and the number of H-bonds to surface sites in order to calculate the pK_a _values of these sites that control surface charging and adsorption. Fitts et al. [38] used second-harmonic generation spectroscopy to show that surface bond lengths and H-bonding arrangements calculated from periodic DFT and classical MD simulations significantly improve the MUSIC-predicted PZC, in contrast to using bulk bond lengths and assumed H-bonding structures. Thus, the structural predictions reported in this study should be useful as input to MUSIC model calculations on charging and adsorption onto α-FeOOH (010) surfaces. The most reliable values are likely to be those determined on the solvated 3 × 3 slab because this model allows for the greatest relaxation and accounts for surface-H_2_O H-bond interactions that will be present in bulk potentiometric experiments.

## Conclusion

Periodic DFT calculations can reasonably reproduce the structures and vibrational frequencies of bulk α-FeOOH. These methods were also applied to predict the surface structures of the (010) α-FeOOH surface. Model predictions of the surface metal-oxygen bond distances and H-bonding configurations should use a simulation cell comprised of multiple surface unit cells to allow for relaxation beyond what is achievable with single unit cell models. Additional layers of H_2_O molecules beyond the L1 layer at metal-oxide surfaces are necessary to reproduce what would be found at the bulk mineral-water interface. The α-FeOOH surface was predicted to have a configuration consisting of FeOH_2_, Fe_2_OH and Fe_3_O groups (i.e., an associated water adsorption model).

## Authors' contributions

JDK performed most of the DFT calculations and was the primary author of the manuscript. KWP performed DFT calculations on the 3 × 3 × 1 supercell of the hydrated surface and determined how to perform the SP-GGA+U calculations. KWP was secondary author of the manuscript, especially with regard to the introduction and methods sections. DLS supervised and reviewed the manuscript throughout its development. All authors read and approved the final manuscript.
